# Three Years with the Army of the Potomac—A Personal Military History

**Published:** 1912-09

**Authors:** William Warren Potter

**Affiliations:** Buffalo, N. Y., Brevet Lieutenant Colonel, U. S. Volunteers; Surgeon in Charge of First Division Field Hospital Second Army Corps; Surgeon 57th Regiment New York Volunteers; Assistant Surgeon 49th Regiment New York Volunteers; Recorder Second Division Hospital Sixth Army Corps, etc., etc.


### Three Years with the Army of the Potomac—A Personal Military History.

By William Warren Potter, M.D., Buffalo, N. Y., Brevet Lieutenant Colonel, U. S. Volunteers; Surgeon in Charge of First Division Field Hospital Second Army Corps; Surgeon 57th Regiment New York Volunteers; Assistant Surgeon 49th Regiment New York Volunteers; Recorder Second Division Hospital Sixth Army Corps, etc., etc.

June 20. We did not fight much yesterday, but this morning heavy cannonading on the right is heard, which probably means that the ball has again opened. We appear to be fighting it out on this line, and I think it very probable that the contest will continue here, even unto the bitter end. The enemy has certainly reached very nearly, if not quite, his last ditch. The weather is very hot, and there is plenty of work to do but very little energy to do it. General Barlow's brother is here and will take a letter from me to Washington, which will enable me to communicate with home.

June 22. Wednesday. Yesterday we moved the hospital about five miles towards the left, and are now about that distance from Petersburg. We crossed the R. R. leading to Suffolk, and are now located at the Smith House near the Jerusalem Plank Road. General Hancock's Headquarters are at the Jones House on the Plank Road; and Headquarters A. of P. are but a few hundred yards away. We are having no fighting today with the exception of a little cannonading on the right, where the Army of the James holds forth. I traded saddles with Captain Elliott while on the march yesterday, and now have a fine English saddle that I like very much better than the one I had before. We are nicely situated again, my tent being in a beautiful yard that surrounds a fine house, and we have plenty of good shade which is a consideration of importance. In an open, out-of-door life like this, the difference between good shade and none at all is simply immense. The house is comfortably furnished, and has such conveniences as we need. I have no idea where the family vanished to, but presume to join the friends of their inclining over in Petersburg. We use the dining-room for our mess, and some of the rooms for the sick officers. Colonel Frank, commanding Third Brigade, is now occupying the parlor, having lost his voice completely, being therefore unable to utter a word of command. Major Kirke died day before yesterday at City Point, and it was found necessary to amputate Captain Wright's foot, so it looks discouraging for him. Captain Middleton has returned to duty, and is in command of the regiment, being the senior officer present. It seems odd to me that Middleton should be in command for when I joined the 57th, he was one of the younger Lieutenants.

June 25. The weather is very hot, and the roads are dusty; but we have a good supply of ice and plenty of lemons, which serve to make life tolerable. Major Hazard, chief of artillery Second Corps, was just here, and I gave him a glass of lemon punch to cool his parched lips. Captain Lewis Nolen and Captain Derrickson were both taken prisoners two days ago.

June 27. Monday. Major Ellis has just been to call upon me, having returned from his Spottsylvania wound. He called to see Mrs. Potter while away, and came to report that all was well at home. He has been appointed Inspector General of the First Division Sixth Corps, and is now on General Russell's staff. Dr. Houston, Dr. Plunkett and Lieutenant Frank Nolen are preparing to leave, as they will be mustered out on the 30th. Dr. Dougherty told me a few days ago that he would make me Surgeon-in-Chief of the Division if my time did not expire so soon; but I informed him that I did not desire the place, as I much preferred my present position. It is more independent, affords me more prefessional employment, and, withal, quite as much honor. Dr. Hall is not more than a quarter of a mile from here with his hospital, and I shall go over and see him this evening. "Brain" Butler was wounded on the 16th or 17th but I did not see him.

June 29. Wednesday. We are still at the Smith House and have had eight days of comparative quietude, which is the longest time we have been in one place since the campaign opened. As Lieutenant Nolen goes out of service tomorrow, I obtained the appointment of Lieutenant Mitchell, of the 57th, in his place as Chief of Ambulances for the Division. I called to see Dr. Hall yesterday, and found him the same as ever, genial and happy. I met Captain Elliot today at Corps Headquarters. Captain Britt has been mustered as Lieutenant Colonel of the 57th, and is back again for duty, though his knee is not yet sound. Lieutenant Hunt of the Artillery, whose foot George L. Potter and I operated on at the Totopotomoy, died in Washington a few days ago. Miss Hancock is at City Point, where large base hospitals have been established, caring for the wounded of the 1st Division.

Saturday, July 2. We are still at the Smith House, the weather is insufferably hot, and as we have had no rain to speak of for a month, the air is full of dust. I have moved my quarters into the house because the dust has become so intolerable. Dr.

Houston will be mustered out to-day, and Dr. Henry A. Martin, U. S. Volunteers, will take his place. Last evening the medical officers of the Division held a meeting here, to give expression to their regrets in thus separating from our old friend and chief. Appropriate resolutions were passed, and a sum of money raised to purchase a suitable testimonial of our regard. Colonel F. A. Walker is again with me, having come over yesterday to have two teeth extracted. He preferred to take chloroform, and has not yet recovered from its secondary effects. Major John Hancock is relieved from duty with the First Division and assigned to the Third, as he could not agree with General Barlow. Walker thinks we will remain here some time.

Monday, July 4. Everything is extremely quiet along the whole line this morning, but two or three guns having been heard, and they far off to the right. Yesterday Dr. Houston, Dr. Plunkett, and Lieutenant Nolen started for home, going off in fine spirits and great glee. I was very sorry to have them go; they were good and faithful officers, and warm friends of mine. Today is much cooler than common here, though we have had no rain worth mentioning, only a little shower last night. The hospital is in fine condition, containing 170 sick patients but, strange to tell, no wounded. The First Division Hospital has the reputation of being one of the best in the Army. Major Ellis called upon me two days ago and was, as usual, looking well.

July 6. Our position remains unchanged, and I hope will continue so for some time, as it is too hot to fight. Mrs. Barlow has been up to see the General, and I took her to City Point at the request of General Barlow, as he could not go with her himself. We went down in the old family carriage found in the barn on the Smith estate, and I saw her safely on board the steamer bound for Washington. She did not seem well but made no complaint at the time. Dr. Martin and Dr. George L. Potter are occupying quarters with me here at the Smith House, and they are both very agreeable companions. It appears that the rebels expected us to attack them on the Fourth of July, and were in readiness for us under arms all day. But we had no idea of making ourselves ridiculous by such nonsense as that on Independence Day. We passed the day very comfortably, while they were sweating with their belts on and their muskets in hand, the hot sun pouring its sweltering rays down upon them. The Third Division Sixth Corps moved somewhere today towards the right, and rumor has it that they go to Harper's Ferry. Captain Wilson came over to see me yesterday, and is looking well. Dr. George L. Potter and I are each sporting a straw hat, and today in riding out to the lines we met General Barlow coming back, who smilingly returned our salute, but never said a word in criticism of our unmilitary head apparel.

Friday, July 8. Colonel Britt is with me now, not having recovered from the injury to his knee received last March. He is applying for leave, and will remain here until it comes. Dr. Wilson, brother of Captain W. and late of the Fifth Corps, committed suicide in Harrisburg a few days ago by cutting his throat. He was a cousin of Dr. George L. Potter, and the news comes through the Philadelphia Inquirer, just received. The Doctor and the Captain are both very much saddened thereby, as they regarded Dr. Wilson with affection.

July 9. Saturday. I visited the 49th, and took supper with Colonel Bidwell and other officers this evening. Major Ellis called while I was there, and we went over to General Russell's Headquarters together. Ellis took me out into the woods to show me a carriage that he had "captured," and while we were looking at it orders came for the Sixth Corps to start for Maryland, to assist in driving out the rebels, who are reported there in force under Early. Ellis was in great glee over the prospect, and flew around to make preparations for the transfer of the troops to their new field; so I hastily bade him good-bye, but never saw him afterwards.

Monday, July 11. We are living in great style at present. Directly across the hall from my parlor is the dining room, where ten officers have their mess, and meet three times a day to enjoy the good things set before them. Dr. George L. Potter occupies one end of the table, and I the other. Our white tablecloth, white china, silver, etc., together with some glassware we have captured, make our table look very homelike and attractive. We have two little negro waiters in white aprons to serve the table, and the whole get-up would do credit to a Broadway restaurant. But it will not last long—such comforts never do, at least in the Army. Captain Wright died in Washington from the effects of his wound and the amputation, and his remains have been sent on to New York. The officers of the 57th have passed appropriate resolutions expressive of our appreciation of him as an officer and a man, and of our sympathy with his family, that have been sent forward to his sisters.

Wednesday, July 13. Night before last we vacated the Smith House, and found ourselves at daylight in the rear of the Fifth Corps Headquarters, where we proceeded to put up the hospital. Today our whole corps moved back also, and is massed in an open field near by, "in reserve." The siege of Petersburg goes bravely on, that is to say, we are pretty nearly as well off as we were three weeks ago, saving the few hundred men we have lost. The Army is greatly delighted at receiving the news of the destruction of the rebel cruiser "Alabama" by the "Kearsarge," off the coast of France last month.

July 15. Friday. I took a ride this morning in company with Drs. Dougherty and George L. Potter, and Captain Pelton, down to the wagon camp about five miles distant, where we saw two men hung for an unmentionable crime. It was a very aggravated case, and of course the punishment was just. Today the weather is much cooler, and we are all delighted with the change. An order has been issued authorizing the employment of acting staff surgeons, from among those regimental surgeons of two or more years' experience and service, whose terms are now expiring. These medical officers are to have the same pay and allowances as regimental surgeons, and are to be allowed one Government horse in addition. This may serve to retain the services of a few who would go out of the field altogether, but I do not believe any considerable number will accept the offer; for they will really have no actual rank, and will simply be employed as any civil physician under contract.

July 18. Monday. We moved about one and a half miles on Saturday, and are now occupying the grounds of the Burchard Mansion on the Norfolk and Petersburg stage road, about a mile east or in rear of General Meade's Headquarters. The plantation is owned by Mr. Burchard, who is the father-in-law of the rebel General Dearing, of the cavalry service. It is well furnished, has a fine Chickering piano with Aeolian attachment, and Mrs. Burchard, with two daughters, attended by some old servants, are occupying the house. Mr. Burchard, himself, went to Petersburg to take Mrs. F. Dearing the day our forces came up, June 16, and our lines have cut off his return, (he did not reach home until the war was over, ten months afterwards.) Dr. Gesner, who went home on sick leave when we were at the Tyler House, has had forwarded to him an extension of his leave for twenty days more. Dr. Rizer, 72d Pa., has also been home for twenty days since the campaign opened. Surely, these be fortunate soldiers. Colonel Walker and Captain Pelton spent this evening with us, and seemed to enjoy their visit very much. Colonel Britt goes home tomorrow on twenty days' leave. A large number of recruits has been sent to the 57th, which may necessitate my remaining in the service longer than I had anticipated. My time will properly expire on September 16, but if the regiment is filled up to any considerable extent, it is probable that I shall stay a month or two longer. I sent money home early in the month and, as I have not yet heard from it, I am apprehensive the rebels have captured it, in a raid they made on the R. R. in Maryland.

July 21. The air is delightfully pure this morning, as we had rain yesterday and the day before. I have heard from home today that the money I sent was received in safety. I have, until lately, been hoping to reach home by September 20, but it looks now as though I would not be able to go until a month later. The Government is beginning to appreciate the services of experienced medical officers, and will hold on to them as long as possible, the difficulty in replacing them being so very great.

Saturday, July 23. I paid a visit to City Point yesterday, and took a look at the hospitals there. They are not in better condition than mine, while their facilities for their preparation and maintenance are far superior. I saw Miss Hancock there and took dinner with her. We have a new thing in the way of concentrated food called condensed egg, the eggs being first dried and then put up in hermetically sealed cans, the same as milk. They are very nice indeed, serving the purpose in the preparation of puddings, custards, and many other dishes, nearly or quite as well as fresh eggs. It is thought a general bombardment of Petersburg with a hundred siege guns, will take place within the next few days.

July 24. We have received orders to move the hospital immediately, a part of the train to go to City Point. The weather is very hot, on that account, I dread the march very much indeed.

July 25. Last night we were refreshed with a delightful rain, which continued all night long. We did not move today as ordered yesterday, so I took a ride this afternoon, making the tour of the various headquarters—Brigade, Division and Corps,—and met Colonel Walker, Captain Favill, and many others of my friends. Captain F. tells me that his Company's time will expire on August 12, and that he willl, therefore, be mustered out on that date. Captain Brownson, mustering officer at Corps Headquarters, informs me that I cannot be mustered out until October 18. Considerable heavy firing is heard all along the lines tonight from heavy mortars and rifled cannon, and it is expected a terriffic bombardment will take place within a day or two.

The organization of the hospital is very complete now. I have one surgeon in charge of food and shelter, whose duty is to superintend putting up the tents, the preparation of the food, beds, etc.; one surgeon as executive officer, who promulgates the orders and attends to their execution; one assistant surgeon as Recorder, who attends to the registering of names, preparation of reports, and all office details; and three prescribing surgeons, who look after the treatment of the sick in the wards; and these, together with hospital stewards, wardmaster, clerk, nurses, cooks, etc.; constitute quite a staff and corps of assistants. The personnel of the staff is as follows:

Surgeon W. W. Potter, 57th N. Y., in charge of Hospital,
Surgeon C. S. Hoyt, 39th N. Y., Executive Officer.
Surgeon Jas. E. Pomfret, 7th N. Y. H. Art'y, Food and Shelter,
Assist.-Surgeon J. C. Norris, 81st Pa., Recorder,
Surgeon M. C. Rowland, 61st N. Y., Chief Prescriber,
Two Assistant-Surgeons to aid the latter.

Dr. George L. Potter has been Surgeon-in-Chief of the Artillery Brigade, and Major John G. Hazard is now Chief of Artillery of the 2d Corps. Dr. Rowland, who was my assistant all winter at Brandy Station, is back again to take charge of the prescribing department of the hospital, and I was glad to obtain the services of so competent and experienced an officer. For dinner today we had roast beef, nice boiled potatoes, fresh butter, good bread, and tomatoes, the latter obtained fresh from Norfolk. For dessert: lemon and cranberry pie, water melon, and ice-cream. Hours for meals: breakfast at eight o'clock; luncheon at twelve-thirty, and dinner at six.

